# The Hippo pathway: a renewed insight in the craniofacial diseases and hard tissue remodeling

**DOI:** 10.7150/ijbs.63305

**Published:** 2021-09-24

**Authors:** Jun Chen, Jingyi Cheng, Cong Zhao, Boxuan Zhao, Jia Mi, Wenjie Li

**Affiliations:** 1 Xiangya School of Stomatology, Central South University, Changsha 410008, China.; 2 Xiangya Stomatological Hospital, Central South University, Changsha 410008, China.; 3 Hunan Key Laboratory of Oral Health Research, Hunan 3D Printing Engineering Research Center of Oral Care, Hunan Clinical Research Center of Oral Major Diseases and Oral Health, Central South University, Changsha 410008, China.; 4 National Key Laboratory of Science and Technology on High-strength Structural Materials, Central South University, Changsha 410083, China.; 5 State Key Laboratory of Powder Metallurgy, Central South University, Changsha 410083, China.

**Keywords:** Hippo signaling pathway, YAP, TAZ, periodontal diseases, oral submucous fibrosis, craniofacial diseases

## Abstract

The Hippo pathway plays an important role in many pathophysiological processes, including cell proliferation and differentiation, cell death, cell migration and invasion. Because of its extensive functions, Hippo pathway is closely related to not only growth and development, but also many diseases, including inflammation and cancer. In this study, the role of Hippo pathway in craniofacial diseases and hard tissue remodeling was reviewed, in attempting to find new research directions.

## Introduction

**Figure 1:**
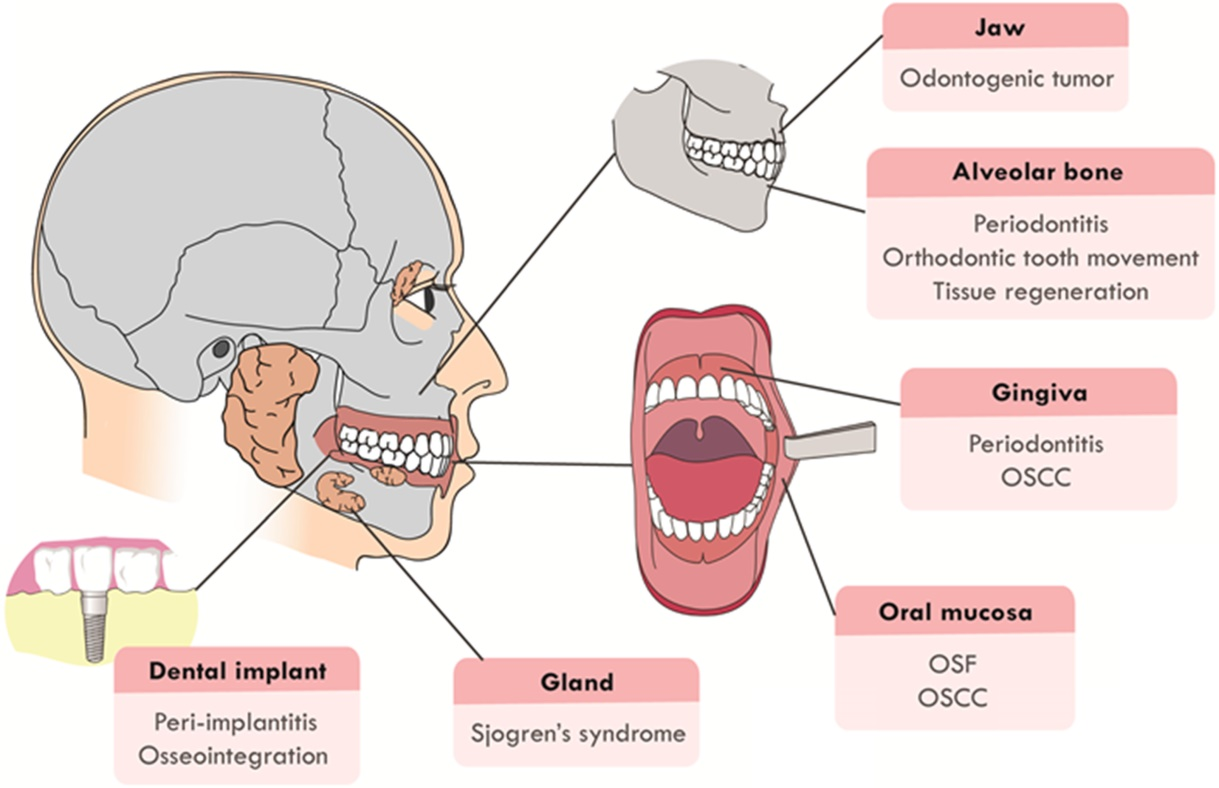
** The craniofacial diseases and hard tissue remodeling that Hippo pathway is involved in**. Hippo pathway plays a role in craniofacial diseases, including OSF, OSCC, odontogenic tumors, periodontitis, peri-implantitis, Sjogren's syndrome and periodontal hard tissue remodeling.

Recently, the function of Hippo signaling pathway has attracted much attention in the craniofacial development and pathological processes. It has been proven that Hippo pathway is involved in the craniofacial inflammatory, cancerous and developmental diseases, such as periodontitis, odontogenic tumors, oral squamous cell carcinoma (OSCC) and tumor drug resistance. Hippo pathway also regulates the remodeling and development of craniofacial bone, the dysregulation of which will cause craniofacial deformity. This review will focus on summarizing recent advancements in two main sections: (1) Hippo pathway in the diseases of the craniofacial region, including oral submucous fibrosis (OSF), OSCC, odontogenic tumors, periodontitis, peri-implantitis and Sjogren's syndrome; (2) Hippo pathway in hard tissue remodeling, including periodontal tissue regeneration and orthodontic tooth movement (Figure 1). An overview of the specific upstream regulators and molecular downstream targets in these processes is also provided.

## An overview of Hippo pathway

**Figure 2:**
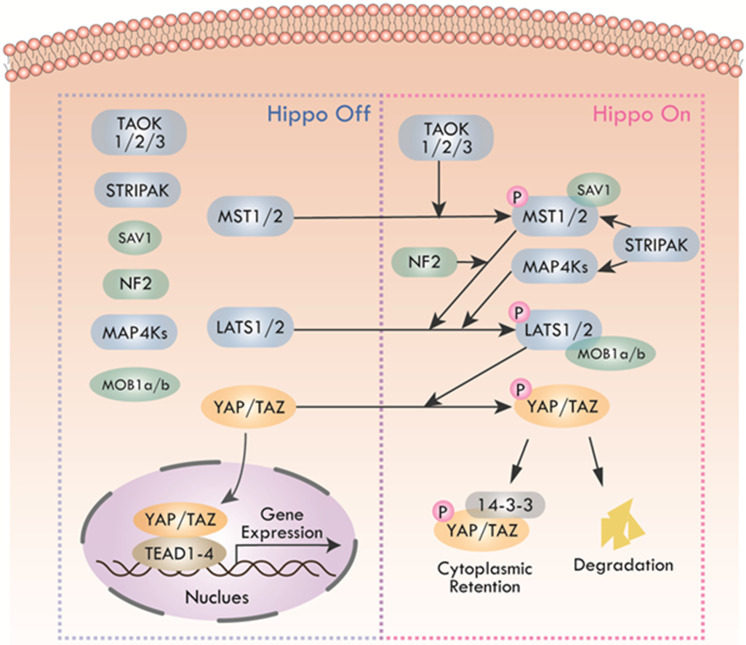
** Canonical Hippo pathway in mammals**. When Hippo pathway is inactivated, YAP and TAZ are dephosphorylated and activated, entering the nucleus and binding with TEAD1-4 to activate downstream gene transcription. Hippo pathway, triggered by TAO kinase, phosphorylates MST1/2 with the help of scaffold proteins SAV1, MOB1a/b and NF2, thus affecting the phosphorylation of LATS1/2. MAP4Ks play similar roles with MST1/2. STRIPAK integrates upstream signals to activate MST1/2 and MAP4Ks. Activated LATS1/2 phosphorylate YAP/TAZ, leading to YAP/TAZ cytoplasmic retention by 14-3-3 protein or degradation.

The Hippo pathway was first caught sight in *Drosophila* melanogaster by genetic mosaic screens for identifying growth suppressors 1, 2. The key compositions of the pathway were highly conserved from *Drosophila* to mammals. Main components of canonical Hippo pathway in mammals include Mammalian Ste20-like protein kinase 1/2 (MST1/2, also known as STK4 and STK3), Salvador family WW domain-containing protein 1 (SAV1), Large tumor suppressor 1/2 (LATS1/2), MOB kinase activator 1A/B (MOB1a/b), and Yes-associated protein (YAP)/Transcriptional co-activator with PDZ binding motif (TAZ) 3-5. The core of Hippo pathway is the kinase cascade, which could be initiated by not only TAO kinases (TAOK1/2/3) that phosphorylated the activation loop of MST1/2, but also the autophosphorylation of MST1/2 through dimerization 6, 7. The activated MST1/2 then phosphorylated SAV1 and MOB1a/b to phosphorylate and activate LATS1/2 8-10. Additionally, recent studies found that mitogen-activated protein kinase kinase kinase kinase (MAP4K) and Merlin (also named NF2) were also activators of LATS1/2 11, 12. MAP4Ks directly activated LATS1/2, while NF2 promoted the phosphorylation of LATS1/2 through MST1/2-SAV complex. The striatin-interacting phosphatase and kinase (STRIPAK) was recently reported to integrate upstream signals to activate MST1/2 and the MAP4Ks 13, 14. Subsequently, activated LATS1/2 could phosphorylate transcriptional co-activators YAP and TAZ, the phosphorylation of which led to sequestration of YAP/TAZ in the cytoplasm by binding with 14-3-3 protein 15. Phosphorylated YAP/TAZ detained in the cytoplasm finally were degraded by recruiting E3 ubiquitin ligases or autophagy 16, 17. Hence, the purpose of the Hippo kinase cascade's terminal physiological output is to restrict the transcriptional activity of YAP/TAZ by preventing YAP/TAZ from entering the nucleus. When the pathway was inactive, unphosphorylated YAP/TAZ accumulated in the nucleus and bounded the transcriptional enhancer associate domain transcription factors (TEADs, including TEAD1-4. Each TEAD had tissue-specific functions during development, such as cardiogenesis 18, neural development 19, myogenesis 20 and trophectoderm lineage determination 21) to promote the expression of target genes such as CTGF, CYR61, ANKRD1, Col8a1 and etc. 22, facilitating cell proliferation, survival, and migration (Figure 2). In recent years, with the deepening of research, Hippo pathway has expanded into a complex signal network with more than 30 components. YAP and TAZ are considered as the canonical effectors of the pathway, responding to various intrinsic and extrinsic signals.

The canonical Hippo pathway regulates numerous biological processes, mainly including cell proliferation and apoptosis. Knockout of MST1, MST2, SAV1 or YAP could lead to liver overgrowth and hepatocellular carcinoma by regulating hepatic cell proliferation 23, 24. MST1 has been considered as a pro-apoptotic kinase, which stimulated apoptosis by phosphorylating Beclin1 and consequently promoting sequestration of Bcl-2 and Bcl-xL by Beclin1 to activate Bax 25. YAP could enhance P53 deacetylation, thus promoting cell survival by repressing P53-induced G0/G1 arrest and apoptosis 26. YAP/TEADs were also associated with the epithelial-to-mesenchymal transition (EMT) transcription factor SLUG to directly inhibit pro-apoptotic BMF, repressing drug-induced apoptosis 27. Some direct target genes of YAP/TAZ, such as Diap1 and BIRC3, have been identified as inhibitors of apoptosis 28. In terms of mechanism, YAP could interact with and stabilize p73 to induce the apoptosis in DNA damage 29. We summarized biological processes and diseases regulated by Hippo pathway, as shown in Table 1.

In addition to the canonical pathway of regulating cell proliferation and apoptosis by inhibiting the entry of downstream transcriptional co-activator YAP into the nucleus, more and more studies have shown that Hippo pathway, with MST1/2 kinase as the core, has a variety of non-canonical regulatory functions. MST1 could mediate oxidative-stress-induced cell death by directly phosphorylating FOXO1/3 to promote FOXO1/3 nuclear translocation in neurons 30, 31. MST1/2 could also enhance FOXO1/3 stability through phosphorylation to promote the development of regulatory T cells and thus inhibit autoimmunity 32. MST1 could directly phosphorylate IRF3, a key transcriptional factor of the antiviral-sensing pathway to inhibit the activation of IRF3 homodimerization and IRF3-mediated transcriptional responses. MST1 could also attenuate IRF3 activation by hindering virus-induced activation of TANK-binding kinase 1 (TBK1), thereby impeding cellular antiviral responses 33. The studies suggest a negative regulation of MST1 in autoimmunity and host defense through non-canonical pathway.

Mechanical signals, stress signals and cell polarity are upstream signals to regulate Hippo pathway 34, 35. On the one hand, YAP/TAZ were proven to play a role in cell type-specific differentiation of mesenchymal stem cells (MSCs) induced by extracellular matrix (ECM) stiffness. ECM stiffness mimicking the natural bone environment could promote the expression of YAP to induce the osteogenic potential of MSCs and enhance the ability of osteogenic differentiation, while soft ECM induced MSCs to differentiate into other cell types, such as adipocytes. Rho-GTPases and the actin cytoskeleton were essential for the nuclear localization of YAP/TAZ during the mechanotransduction linked with ECM stiffness 36. High cell density could also lead to the growth inhibition of cells through Hippo pathway 15. Activation of Hippo kinase cascade in high cell density was associated to the trans-dimerization of E-cadherin at adherens junctions (AJs) 37, as well as the angiomotin (AMOT) complex in the tight junctions (TJs) 38. On the other hand, Hippo pathway could respond to internal and environmental stressors that interfere with the normal state. When cells were faced with energy deficiency, the cellular energy stress led to increased AMP/ATP ratio, which activated the energy sensor AMP-activated protein kinase (AMPK). Activation of AMPK activated LATS1/2 or directly phosphorylated YAP, thus disrupting YAP-TEADs interaction and restoring energy homeostasis 39, 40. Other stress signals, such as oxidative stress 41 and cytokinesis failure 42 were also proved to activate Hippo signaling. It has been proved that cell polarity and adherence signals could activate the Hippo pathway. Many cell polarity components (Mer-Ex-Kibra complex, the apical transmembrane protein Crumbs, the Fat-Dachsous complex, etc.) have been demonstrated to regulate YAP/TAZ activity through Hippo pathway 34. The Hippo signaling could also be initiated by components of junction complexes, including α-catenin, tyrosine-protein phosphatase non-receptor type 14 (PTPN14) and E-cadherin 43.

## Hippo pathway in the craniofacial diseases

### Hippo pathway in oral precancerous lesions

OSF is an oral precancerous lesion characterized by abnormal collagen deposition, closely related to betel nut chewing and commonly found in the Hunan Province of mainland China, India and Southeast Asia 44. In arecoline treated endothelial cells, YAP was up-regulated with an enhanced transcriptional activity, which induced the activation of EMT. Abnormal deposition of Type I and III collagen is an important pathological feature of OSF, and knocking down of YAP can inhibit the secretion of Type I and II collagen. YAP-induced EMT was considered as a crucial event in OSF 45. It has been proven that knockdown of bone morphogenetic protein 4 (BMP4) could affect the expression of EMT markers and inhibit ECM accumulation. Studies have shown that BMP4 as the downstream mediator was induced by the activation of YAP, which induced EMT in OSF 46. The role of YAP on EMT in OSF suggested the potential target therapeutic value of YAP. The mechanical stimulation of oral mucosa caused by betel nut chewing also could lead to YAP activation, which promoted keratinocyte proliferation in OSF 47. The mechanical activation of YAP/TAZ caused by increased matrix stiffness in tissue fibrosis could promote proliferation and survival of epithelial cells, which is crucial to the malignant transformation of OSF 47, 48. However, the function of other components of Hippo pathway in the development of OSF, and the exact mechanism of Hippo pathway in the pathogenesis of OSF still need further elucidation. Until now, there are few studies on the role of Hippo pathway in other oral precancerous lesions such as oral leukoplakia, erythema and lichen planus. The relevant mechanisms remain to be supplemented, which may provide new clues for targeted therapy and recurrence prevention of oral premalignancy diseases.

### Hippo pathway in OSCC

Hippo signaling pathway is known for regulating the occurrence and development of cancer. Knockout of MST1, MST2, SAV1 or YAP could lead to liver overgrowth and hepatocellular carcinoma 23, 24. MST1, MST2 or SAV deletion could activate YAP to increase the intestinal crypt proliferation and tumorigenicity 49. Studies of human colorectal cancer also have shown that the YAP/TAZ were closely related to tumor growth 50. In addition, YAP/TAZ could inhibit apoptosis in the development of various cancers, such as breast cancer and non-small-cell lung cancer 51, 52. A few direct target genes of YAP/TAZ, such as Diap1 and BIRC3, have been identified as inhibitors of apoptosis 28. Collectively, Hippo pathway is closely related to carcinogenesis due to uncontrolled cell proliferation and apoptosis. Additionally, stromal stiffness in solid tumor tissues activated Hippo pathway as a type of mechanical signals. YAP has been proven to be activated in cancer-associated fibroblasts (CAFs) in response to mechanical stress and perturbation of the actin cytoskeleton, which established a positive-feedback loop to maintain the CAF phenotype 53.

Recently, the role of Hippo pathway in OSCC has also attracted attention. TP53, FAT1, PTEN, and EGFR were identified as upstream regulators of YAP, whereas TP63 was considered as a downstream effector of YAP. OSCC patients usually carry mutations in TP53, FAT1, PI3K/PTEN or EGFR signaling. These mutations could improve the activity of YAP in OSCC 54. The YAP hyper-activation has been found to play a role in the early onset and rapid progression of OSCC, promoting tumorigenic phenotypes in OSCC cells and being essential to the development and metastasis of OSCC 55. MiR-130a was highly expressed in OSCC cell lines. Overexpression of miR-130a promoted OSCC cell proliferation, metastasis and invasion by inactivating the Hippo signaling and activating YAP 56. YAP could also be triggered by the over-expression of phosphatidylinositol 3-kinase catalytic subunit alpha (PIK3CA) 57, one of the most common oncogenic events in multiple malignancies including OSCC 58. One study reported that Piezo-type mechanosensitive ion channel component 1 (PIEZO1), a Ca2+ channel, was a transcriptional target of YAP, regulating OSCC cellular growth 59. TAZ has been revealed to promote the proliferation, migration, invasion, EMT and cancer stem cell maintenance of OSCC cells 60. Studies have shown that TAZ enhanced the self-renewal and maintenance of cancer stem cells (CSCs) by directly transcriptional activating downstream sex determining region Y box 2 (SOX2), the key transcriptional factor regulating CSCs properties from diverse cancer origins including OSCC 61. Chronic mechanical damage of oral mucosa caused by long-term denture stimulation was a risk factor of OSCC. A recent study suggested that Hippo pathway acted as a mediator between denture-induced mechanotransduction and carcinogenesis. In the masticatory movements of denture wearer patients, mechanical stresses (shear, compressive, and tensile) transferred directly to palatal and alveolar mucosa, which was regulated by Hippo pathway and caused subsequent YAP/TAZ activation, promoting the symbolic biological behavior of cancer such as proliferation, survival, invasion, migration and angiogenesis 62. In a word, the cascade reaction of Hippo pathway could exert a vital function in the onset and progression of OSCC.

Hippo pathway also plays a role in drug resistance of some cancers. Studies have shown that when YAP/TAZ were activated, tumor cells acquire the ability of chemotherapeutic drug resistance. TAZ could sustain the survival of breast cancer stem cells when treated with conventional chemotherapeutics such as paclitaxel and doxorubicin 63. In addition, YAP/TAZ activation protected multiple tumor types from DNA damaging agents, including cisplatin, UV, and radiation 64-66. YAP could also make tumor cell lines with BRAF, KRAS or NRAS mutations resistant to RAF and MEK inhibitor therapy 67. Recently, the role of YAP on the drug resistance of OSCC has also been explored. Activation and nuclear translocation of YAP contributed to the acquisition of cisplatin resistance in OSCC 68. One study showed that Ribosomal binding protein 1 (RRBP1) induced cisplatin resistance in OSCC by up-regulating YAP expression 69. YAP over-expression led to gefitinib resistance in OSCC cells 70. YAP has been suggested as a biomarker of treatment response in OSCC 70, 71.

Furthermore, studies have shown YAP/TAZ activation was associated with poor prognosis for OSCC. YAP activity was correlated with malignant phenotypes and poor prognosis 55, 72. Pyruvate kinase M2 (PKM2), the key rate‑limiting enzyme of glycolysis, highly expressed in oral tongue squamous cell carcinoma (OTSCC) tissues, maintaining the Warburg effect in tumor cells. The higher PKM2 expression was demonstrated to be related with a higher Tumor‑Node‑Metastasis (TNM) stage and a shorter overall survival. PKM2 knockdown could inhibited the proliferation and increased the apoptosis of OTSCC cells by activating Hippo signaling pathway, as confirmed by the decreased expression of YAP 73. TAZ over-expression was also relevant to higher pathological grade, lymph node metastasis and poor prognosis 60, 74.

Due to the important role of YAP/TAZ in the pathogenesis, drug resistance and prognosis of OSCC, target for the components of Hippo pathway may probably bring new therapies for OSCC.

### Hippo pathway in odontogenic tumors

Odontogenic tumor is a kind of tumor derived from odontogenic tissue, which includes enamel organ, dental sac and dental papilla. Keratocystic odontogenic tumor (KCOT) has been reported to be the most common odontogenic tumors in China 75, with some particular histologic features like parakeratinized stratified cell layers and daughter cysts 76. In KCOT, YAP/TAZ were involved in the pathogenesis and proliferative growth. Compared with normal tissues, YAP/TAZ and downstream proteins (CYR61 and CTGF) were significantly up-regulated in KCOT 77. In KCOT, the crosstalk between YAP/TAZ and Ki-67 has also been reported, revealing the Hippo signaling-mediated proliferative behavior 77. In addition, YAP was over-expressed in odontogenic epithelium of ameloblastoma compared with the epithelial islands of dental follicle. YAP was considered to be essential for the neoplastic nature of ameloblastoma, and contribute to tumor invasiveness 78. Although emerging studies have suggested the Hippo pathway was involved in odontogenic tumors, the exact mechanism still needs further investigations.

### Hippo pathway in periodontitis

In recent years, more and more researches have confirmed that Hippo pathway was involved in the regulation of inflammation. Hippo pathway plays a pro-inflammatory role by inhibiting the activity of YAP, which exerts an anti-inflammatory effect.

Pathogen-associated molecular patterns (PAMPs) are key molecules that trigger inflammation in inflammatory processes, including periodontitis 79, 80. PAMPs could be recognized by multiple pattern recognition receptors (PRRs) like toll-like receptors (TLRs) to activate downstream inflammatory cascades 81, 82. After TLRs recognizing PAMPs, the cytoplasmic Toll/interleukin (IL) 1 receptor (TIR) domains of the TLRs recruit the signaling adaptors, including MyD88, TIRAP, TRAM, and/or TRIF. According to the selection of specific adapter, various kinases (IRAK4, IRAK1, IRAK2, TAK1, TBK1, IKKε, etc.) and ubiquitin ligases (TRAF6, pellino 1, etc.) are activated to activate NF-κB, type I interferon, MAPK and JNK pathways, leading to the expression and release of pro-inflammatory cytokines 81, 82. Inflammatory cytokines, including TNF-α and IL-1β could not only initiate Hippo pathway-induced YAP/TAZ degradation but also TAK1-mediated YAP/TAZ degradation. Furthermore, YAP impeded TAK1 substrate accessibility to prevent the downstream IKKα/β activation, thus inhibiting NF-κB signaling 83.

**Figure 3:**
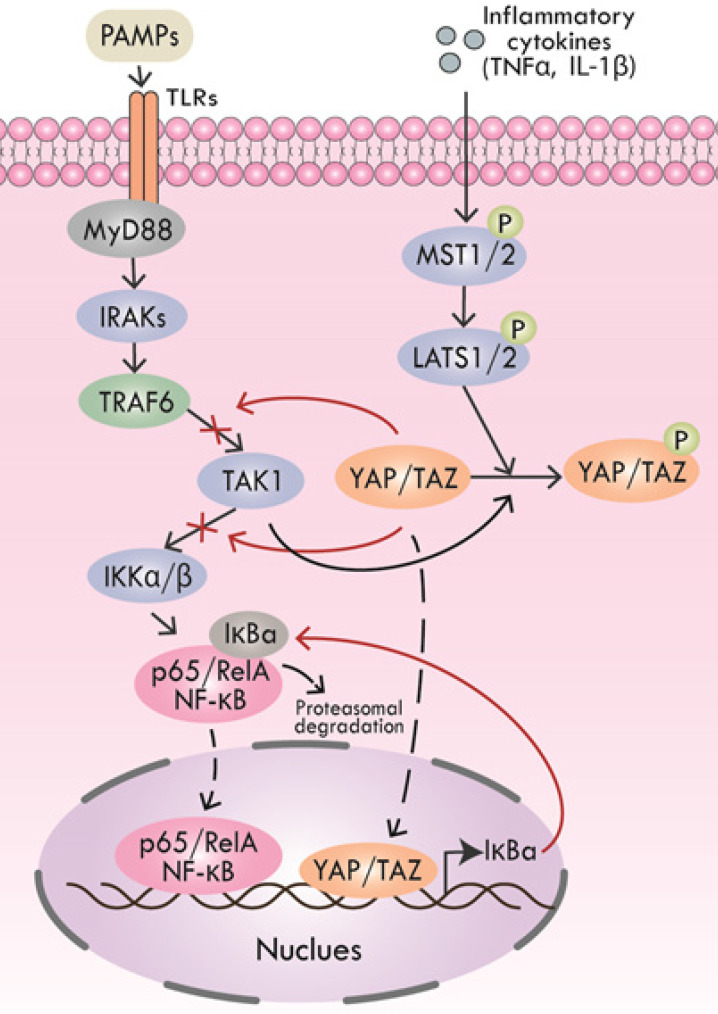
** Regulation of Hippo pathway on inflammatory signals.** YAP and TRAF6 synergistically inhibit the activation of downstream protein TAK1, thereby suppressing NF-κB signaling. YAP also interacts with TAK1 to inhibit downstream IKKα/β activation, thus inhibiting NF-κB signaling. TNF-α and IL-1β initiate YAP/TAZ degradation through Hippo signaling. Inhibitory IκBa expression induced by nuclear YAP/TAZ inhibits NF-κB response.

Lipopolysaccharides (LPS) is a typical kind of PAMPs, which is the cause of many inflammatory diseases, including periodontitis. In the LPS-triggered cascade of mammalian endothelial cells, YAP could directly bind to TRAF6, promote K48 ubiquitination to degrade TRAF6, and inhibit K63 ubiquitination to block the activation of downstream protein TAK1, thereby inhibiting the NF-κB signaling pathway and alleviating inflammatory response 84. Additionally, mice with a lack of YAP/TAZ in the alveolar epithelial type II cells (AEC-IIs) showed extended inflammatory responses during bacterial pneumonia, revealing the anti-inflammatory function of YAP/TAZ. Nuclear YAP/TAZ expression was significantly increased in AEC-IIs infected by Gram-positive pathogen Streptococcus, and then YAP/TAZ-induced IκBa expression was enhanced, which suppressed NF-κB response 85 (Figure 3).

Alveolar bone loss is a hallmark of periodontitis progression 86. Hippo pathway has been reported as a biochemical signal response effector in bone remodeling regulation. Hippo pathway could transform inflammatory stimuli induced by periodontitis into regulation of osteoblasts and osteoclasts activity. NF-κB receptor activator (RANK) and NF-κB ligand receptor activator (RANKL) jointly promote differentiation, maturation and activation of osteoclasts and progenitor cells during inflammatory bone remodeling, while osteoprotegerin (OPG) blocks this process and inhibits osteoclasts differentiation, thereby alleviating bone damage 87, 88. In response to inflammatory stimuli, YAP not only decreased the expression of RANKL, but also enhanced the expression of OPG in osteoblast to resist inflammatory bone resorption 89. Additionally, α-calcitonin gene-related peptide (αCGRP) could up-regulate the expression of osteogenic phenotype in human periodontal ligament cells (hPDLCs) by activating YAP 90. However, one study reported a contrary finding that periodontitis with traumatic occlusion (TO) is a condition in which inflammatory stimuli and mechanical stress signals work together, and that YAP can increase bone resorption, but the exact mechanism remains unclear 91.

Angiogenesis and bone immune are two important events in periodontitis 92, 93. Angiogenesis is the key biological processes in periodontal tissue and organ reconstruction 94. YAP/TAZ have been proved as a key regulator of angiogenesis and vascular barrier maturation by activating actin cytoskeleton remodeling and endothelial cell (EC) proliferation 95. VEGF could activate YAP/TAZ by acting on the actin cytoskeleton, triggering transcriptional programs to control cytoskeleton dynamics, thereby establishing feedforward cycles to ensure appropriate angiogenic response 96. VEGF/VEGFR could also inhibit MST1/2 via PI3K/MAPK to impede Hippo signaling and activate YAP/TAZ 97. These results have indicated that YAP/TAZ acted as downstream effectors of VEGFR. Furthermore, YAP/TAZ were demonstrated to initiate angiogenesis through YAP/TAZ-ANG-2/CYR61 axis 97.

The concept of osteoimmunology was first proposed by Arron et al., declaring the correlation between bone and immune system. Bone cells participate in immune regulation by secreting cytokines. Abnormal activity of immune cells results in changes in osteogenesis and angiogenesis^98^. Macrophage is the major participant in bone immune response. Studies have shown that Hippo-YAP pathway plays a role in the polarization of macrophage to M1/M2 phenotypes. Wnt5a promoted TGFβ1-mediated M2 polarization by inducing YAP/TAZ 99. The inhibition of Hippo pathway in macrophage enhanced not only YAP nuclear translocation but also interaction with β-catenin, which could impede XBP1-mediated NLR family pyrin domain containing 3 (NLRP3) activation, resulting in IL-1β release reduced and M2 macrophage phenotype promoted 100. However, some studies have shown opposite results. For example, YAP was reported to impair M2 but facilitate M1 macrophage polarization. Myeloid YAP knockout resulted in increased M2 phenotypic IL-10 and decreased M1 phenotypic IL-1β 101. αCGRP-YAP signaling was proved to inhibit osteogenic factors secretion of M2 macrophages at first, and then promote the osteogenic factor secretion by regulating the bone immune response of M2 macrophages 102.

As mentioned above, although studies on the role of Hippo-YAP pathway in angiogenesis and bone immunity have been in-depth, there are still few studies on the role of Hippo pathway in regulating periodontitis through angiogenesis and bone immunity. Therefore, studies in this field can be considered in the future. In addition, whether Hippo pathway regulates periodontal ligament regeneration needs further study.

### Hippo pathway in peri-implantitis

Hippo-YAP pathway has also been revealed to be involved in the regulation of peri-implantitis. Titanium (Ti) and its corresponding alloys have been widely used in dental implants. Ti ions can be released from the implant into surrounding tissues in the presence of host detrimental electrolytic aqueous environment 103. In the early stage of Ti exposure, NF-κB pathway was activated by up-regulating the nuclear expression of YAP, triggering an inflammatory response and resulting in tissue damage adjacent to oral implants. In this process, YAP over-expression enhanced Ti-induced inflammatory response through NF-κB pathway. However, the continuous activation of NF-κB pathway inhibited the expression of YAP and thus suppressed the inflammatory response, which acting as a negative feedback^104^. In addition, Ti ions could also inhibit osteogenic differentiation of osteoblasts by increasing YAP nuclear expression 103. Taken together, the promotion of inflammatory response and inhibition of osteogenesis in peri-implantitis induced by Ti ions were both associated with the YAP over-expression.

### Hippo pathway in Sjogren's syndrome

Sjogren's syndrome (SS) is a complex autoimmune disease primarily affecting salivary and lacrimal glands. Although immune deficiency is considered to be the main cause of SS, increasing studies indicate that loss of cell polarity and structural integrity, including E-cadherin adhesion deficiency, play an important role in SS. E-cadherin has been shown to be an important regulator of morphologic transformation during submandibular gland (SMG) development, which could establish the apical-basal polarity in acinar and ductal precursor cells 105. Recently, studies have shown that the E-cadherin junctions can interact with the Hippo pathway, which is necessary for SMG branching morphogenesis. These findings from the mouse model were validated in human labial biopsy specimens from patients with SS 106. The interaction of TAZ with E-cadherin and α-catenin, two major determinants of cell polarity, was essential for normal gland cell differentiation and structure organization 106. Moreover, YAP was involved in the formation of SMG epithelial progenitor cells by inducing Epiregulin expression, and dysregulated YAP-mediated cell fate control was thought to be related to SS 107.

## Hippo pathway in hard tissue remodeling

### Hippo pathway in periodontal tissue regeneration

Periodontal tissue contains alveolar bone, gingiva, periodontal ligament and cementum. The main function of the periodontal tissue is to support, fix and nourish teeth. Periodontal tissue regeneration is of great significance to the treatment of periodontal diseases. Recent studies have shown that Hippo pathway acts as an important regulator in periodontal tissue regeneration.

Human periodontal ligament stem cells (hPDLSCs) are considered as the most promising seed cells for periodontal tissue regeneration owing to their self-renewing and multi-lineage differentiation 108. Activation of YAP could promote the proliferation of hPDLSCs, inhibit cell apoptosis, and delay cell senescence 109, 110. Hippo pathway inactivation and YAP dephosphorylation could promote cell proliferation and inhibit apoptosis and senescence induced by telomerase reverse transcriptase (TERT) overexpression 111. Over-expression of TAZ could promote the proliferation of hPDLSCs and inhibit apoptosis 112. The crosstalk between Hippo and ERK or AKT has been considered as a vital process in periodontal tissue regeneration. When YAP was up-regulated, protein levels of P-Msk1, P-ERK1/2 and its target genes were increased 110, suggesting that YAP could activate ERK pathway. ERK and AKT pathway were involved in various biological processes, including the regulation of cell metabolism, proliferation and survival 113, 114. In hPDLSCs, the phosphorylation of ERK and AKT was affected by YAP, which was responsible for the regulation of the cell proliferation and cell senescence 111. Studies have also found that YAP/TAZ can balance the process of osteogenic and adipogenic differentiation in hPDLSCs. The up-regulation of YAP promoted osteogenic differentiation and inhibit adipogenic differentiation, while knockdown of YAP led to an opposite result. In this process, YAP enhanced the stabilization and nuclear transfer of β-catenin, possibly by regulating upstream proteins of Wnt/β-catenin pathway, including LRP6 and DVL3 115. Over-expression of TAZ also promoted osteogenic differentiation of hPDLSCs through p-SMAD3^112^. We summarized the signaling crosstalk of Hippo pathway in periodontal tissue regeneration, as shown in Table 2.

The regulatory role of YAP in other types of oral stem cells has also been reported. YAP mediated the mineralization induced by the static magnetic field in dental pulp stem cells (DPSCs) 116, 117. MiR-141-3p inhibited proliferation and promoted senescence of stem cells from apical papilla (SCAPs) by post-transcriptionally downregulating YAP 118. Interestingly, some studies have shown inconsistent results in the role of YAP in osteogenesis-related cells 103, 119. Some studies have shown that YAP can inhibit the osteoblastic activity of osteoblasts. The reason is that the WW domain of YAP interacts with the PY motif of Runx2 to form the YAP/ Runx2 complex, thus inhibiting the activity of Runx2 in osteoblasts 119, 120. Similar result of YAP inhibiting the osteogenic differentiation was also showed in mesenchymal stem cells (MSCs) 120. However, physical stimulations could promote osteogenesis by activating YAP 121, 122.

An *in vivo* study of oral implant bone healing showed that αCGRP could up-regulate the expression of YAP and αCGRP-YAP pathway could promote angiogenesis and osteogenesis during implant bone healing, especially in the early stage 123.

As crucial members of Hippo pathway, YAP and TAZ contributed to promoting proliferation, inhibiting apoptosis and senescence, as well as regulating osteogenic or lipogenic differentiation of many types of oral stem cells. Targeting YAP/TAZ to regulate the proliferation and differentiation of hPDLSCs through the Hippo pathway may provide a new strategy for periodontal tissue regeneration, which may be a direction of future research.

### Hippo pathway in orthodontic tooth movement

Mechanical stress, as an important factor, could activate YAP/TAZ to induce osteogenic differentiation of hPDLCs, which contributes to the alveolar bone formation in orthodontic tooth movement (OTM). Cyclic stretch was found to induce YAP nuclear localization and activity, promoting the osteogenic differentiation of hPDLCs 124. In a high-throughput sequencing study, the expression profile of stretched hPDLCs revealed several important components of the Hippo pathway such as LATS1, YAP and TEAD1/2 were up-regulated 125. One study has shown that TAZ adjusted bone tissue remodeling through Runx2, while YAP regulated periodontal cell proliferation and differentiation during OTM 126. In general, Hippo pathway acts as a mechanical-chemical signal transduction hub in OTM, which coordinates bone remodeling and tooth relocation. However, the study of Hippo pathway in OTM is still not in-depth, and future research can be strengthened in this aspect.

## Conclusion

**Figure 4:**
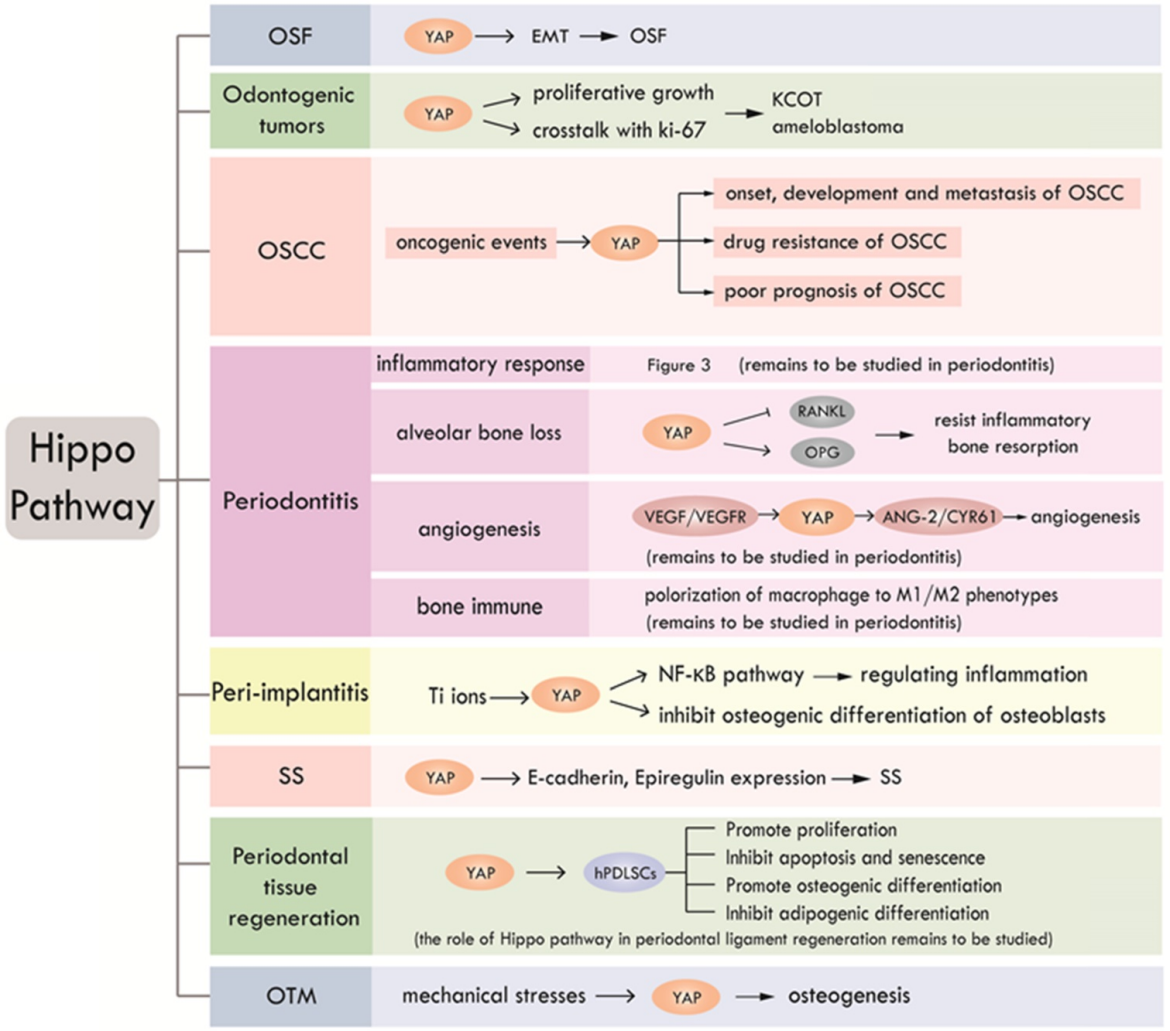
** Research status and prospect of Hippo pathway in craniofacial diseases and hard tissue remodeling.** Hippo pathway is involved in OSF, odontogenic tumors, OSCC, periodontitis, peri-implantitis, SS, periodontal tissue regeneration and OTM through different biological processes.

According to the existing literatures, Hippo pathway plays an important role in a slew of oral events, including periodontal tissue regeneration, periodontitis, peri-implantitis, SS, odontogenic tumors, OSF and OSCC. We summarized the research progress of Hippo pathway in oral diseases, as shown in Table 3 and Figure 4. To date, studies about Hippo pathway in periodontal tissue regeneration were mainly focused on the regulation of osteogenesis in hPDLSCs, while the function of Hippo pathway in periodontal ligament regeneration still needs to be elucidated. The involvement of Hippo pathway in periodontitis by regulating angiogenesis and bone immunity is also worthy of further study. The role of Hippo pathway in peri-implantitis is still in its infancy. What's more, further researches on the role of Hippo pathway in the development of OSF are expected. Mechanism studies on the role of Hippo signaling in OSF and its carcinogenesis will help clarify whether Hippo pathway has the potential to be a new therapeutic target for OSF. Further researches can also focus on investigating the function of Hippo pathway in other oral precancerous lesions such as oral leukoplakia and erythema.

To sum up, Hippo pathway is involved in various physiological and pathological processes of craniofacial diseases, a thorough study of which may help to better reveal the occurrence and development of craniofacial diseases and provide new ideas for the treatment.

## Figures and Tables

**Table 1:** Biological processes and diseases regulated by Hippo pathway

Biological processes regulated by Hippo pathway	Related diseases	Reference
Cell proliferation	Liver overgrowth, Hepatocellular carcinoma, Colorectal cancer, Hypertrophic cardio-myopathy	23, 24, 50, 127-129
Cell cycle	OSCC, Pancreatic cancer	55,130
Cell apoptosis	Breast cancer, Non-small-cell lung cancer, Hematological malignancies	51,52,131
Cell autophagy	Aggressive breast cancers, Hepatocellular carcinoma, Thyroid cancer	132-134
Cell senescence	Breast cancer, Osteoarthritis	135,136
EMT	Organ fibrosis and cancer (lung, kidney, skin, liver)	48
Angiogenesis	Cholangiocarcinoma, Pancreatic ductal adenocarcinoma	137,138
Drug resistance of tumor cell	Breast cancer, Colorectal cancer, Esophageal cancer	63,139,140

**Table 2:** Signaling crosstalk of Hippo pathway in periodontal tissue regeneration

Signaling crosstalk of the Hippo signaling pathway	Related Cellular Processes	Reference
Wnt/β-catenin	Differentiation	115, 141, 142
ERK	Proliferation, Apoptosis, Senescence	109-111
PI3K/AKT	Proliferation, Senescence	110, 112
TGF-β/SMAD	Proliferation, Differentiation	112

**Table 3:** The role of Hippo pathway in oral diseases

Oral Disease	Upstream Signals	The effector in Hippo pathway	Activated/Inhibited	Effect	Reference
OSF	arecoline	YAP	Activated	Promote EMT	45
	mechanical stresses			Promote keratinocyte proliferation	47
OSCC	TP53/FAT1/PTEN/EGFR/MiR-130a/PIK3CA	YAP	Activated	Promote proliferation, metastasis, invasion, etc.	54, 56, 57
	mechanical stresses				62
	-	TAZ			60
	RRBP1	YAP	Activated	Promote cisplatin resistance	69
	-			Promote gefitinib resistance	70
	PKM2	YAP	Activated	Promote the poor prognosis	73
	-	TAZ			60, 74
Odontogenic tumor	-	YAP/TAZ	Activated	Promote proliferation, invasion, etc.	77, 78
Periodontitis	-	YAP	Activated	Resist inflammatory bone resorption	89
Peri-implantitis	Ti ions	YAP	Activated	Promote inflammatory responses; Inhibit osteogenic differentiation of osteoblasts	103, 104
SS	-	YAP/TAZ	Activated	Promote gland formation	105-107
Periodontal tissue regeneration	-	YAP	Activated	**In hPDLSCs:** Promote proliferation; Inhibit apoptosis and senescence; Promote osteogenic differentiation; Inhibit adipogenic differentiation	109, 110, 115
Implant osseointegration	αCGRP	YAP	Activated	Promote angiogenesis and osteogenesis	123
Orthodontic tooth movement	mechanical stresses	YAP	Activated	Promote osteogenic differentiation of hPDLCs	124

## Abbreviations

OSCC: oral squamous cell carcinoma
OSF: oral submucous fibrosis
MST1/2: mammalian Ste20-like protein kinase 1/2
SAV1: salvador family WW domain-containing protein 1
LATS1/2: large tumor suppressor 1/2
MOB1a/b: MOB kinase activator 1A/B
YAP: yes-associated protein
TAZ: transcriptional co-activator with PDZ binding motif
MAP4K: mitogen-activated protein kinase kinase kinase kinase
STRIPAK: striatin-interacting phosphatase and kinase
TEADs: transcriptional enhancer associate domain transcription factors
EMT: epithelial-to-mesenchymal transition
ER: endoplasmic reticulum
TBK1: TANK-binding kinase 1
MSCs: mesenchymal stem cells
ECM: extracellular matrix
AMOT: angiomotin
AMPK: AMP-activated protein kinase
PTPN14: tyrosine-protein phosphatase non-receptor type 14
BMP: bone morphogenetic protein
CAFs: cancer-associated fibroblasts
PIK3CA: phosphatidylinositol 3-kinase catalytic subunit alpha
CSCs: cancer stem cells
SOX2: sex determining region Y box 2
PAMPs: pathogen-associated molecular patterns
PRRs: pattern recognition receptors
TLRs: toll-like receptors
TIR: Toll/interleukin (IL) 1 receptor
IL: interleukin
AECIIs: alveolar epithelial type II cells
RANK: NF-κB receptor activator
RANKL: NF-κB ligand receptor activator
OPG: osteoprotegerin
αCGRP: α-calcitonin gene-related peptide
hPDLCs: human periodontal ligament cells
TO: traumatic occlusion
EC: endothelial cell
NLRP3: NLR family pyrin domain containing 3
SS: Sjogren's syndrome
SMG: submandibular gland
hPDLSCs: human periodontal ligament stem cells
TERT: telomerase reverse transcriptase
DPSCs: dental pulp stem cells
SCAPs: stem cells from apical papilla
MSCs: mesenchymal stem cells
OTM: orthodontic tooth movement
